# Identifying Drug Combination Strategies for *ZMYM2: FGFR1* Fusion Positive Leukemia

**DOI:** 10.1080/28354311.2025.2530229

**Published:** 2025-07-16

**Authors:** Ariane Huang, Sofia R. Beer, Christopher A. Eide, Brian J. Druker, Jeffrey W. Tyner, Jessica Leonard, Cristina E. Tognon

**Affiliations:** a Knight Cancer Institute, Oregon Health & Science University, Portland, OR, USA; b Chicago Stritch School of Medicine, Loyola University, Maywood, IL, USA; c Division of Hematology & Medical Oncology, Department of Medicine, Oregon Health & Science University, Portland, OR, USA; d Department of Cell, Developmental, and Cancer Biology, Oregon Health & Science University, Portland, OR, USA; e Division of Oncological Sciences, Department of Medicine, Oregon Health & Science University, Portland, OR, USA

## Abstract

Myeloid/lymphoid neoplasms with eosinophilia and tyrosine kinase gene fusions (MLN-TK) are a class of fusion protein-driven, poor prognosis leukemias. Leukemias harboring FGFR1 fusions have previously been referred to as 8p11.2 myeloproliferative syndrome (EMS) or stem cell leukemia/lymphoma (SCLL) and are currently referred to as Myeloid/lymphoid neoplasms with FGFR1 rearrangement based on the most recent WHO classification system. To identify new therapeutic options for MLN-TK patients, we evaluated clinical and *ex vivo* drug response data from a *ZMYM2:FGFR1*-positive patient who was successfully treated with FGFR kinase-targeting inhibitors. After initially responding to ponatinib, the patient was switched to pemigatinib which eventually transitioned them to a successful transplant. Leukemia cells isolated from the patient exhibited ex vivo sensitivity to ponatinib, bortezomib and axitinib. ZMYM2:FGFR1-transformed Ba/F3 cells were exquisitely sensitive to next generation FGFR inhibitors, and combinations of FGFRi plus trametinib or midostaurin were found to be synergistic, suggesting novel therapeutic options for FGFR1-fusion positive patients.

The World Health Organization (WHO) classifies myeloid/lymphoid neoplasms with eosinophilia and tyrosine kinase gene fusions (MLN-TK) as those driven by translocation of genes such as PDGFRA/B, JAK2, FLT3, ABL1 or FGFR1.^1^ MLN-TK neoplasms are highly aggressive and feature increased risk for progression to acute leukemia. Leukemias harboring FGFR1 fusions have historically been referred to as 8p11.2 myeloproliferative syndrome (EMS) or stem cell leukemia/lymphoma (SCLL).^2,3^ The latest World Health Organization (WHO) classification now refers to these diseases as Myeloid/lymphoid neoplasms with FGFR1 rearrangements (MLN-FGFR1^1^). Fourteen FGFR1 fusion partners have been identified to date, with ZMYM2 (Zinc-finger domain ZNF198) on chromosome 13q12.11 being the most common. Multiple FGFR kinase inhibitors are currently being evaluated clinically for MLN-FGFR1 leukemias.^4^ Despite these advances, drug resistance and toxicity remain a treatment limitation, motivating additional work to address these challenges.

In this report we describe the case of a patient with a ZMYM2:FGFR1 fusion-driven leukemia who was successfully treated with FGFR kinase-targeting inhibitors. A 36-year-old man presented with fatigue, weight loss and cervical adenopathy, laboratory findings showed leukocytosis (WBC = 88,000/uL) with mild anemia and thrombocytopenia. Bone marrow biopsy (BMBx) revealed a hypercellular marrow (95%) with marked myeloid hyperplasia and mild megakaryocytic atypia, 2–3% blasts, absolute eosinophilia, and mild absolute basophilia, not consistent with acute leukemia. However, fluorescence in situ hybridization (FISH) confirmed the diagnosis of Myeloid/lymphoid neoplasms with FGFR1 rearrangements based on the t (8;13)(p11; q12) translocation (Figure 1A, resulting in the *ZMYM2: FGFR1* fusion gene that was confirmed by Sanger sequencing (Figure 1B; Day 1 in the timeline Figure 1C). Clinical exome capture library (GeneTrials) sequencing revealed a *RUNX1*^R201Q^ mutation (Variant Allele Frequency (VAF): 41%) at diagnosis and the disease remained clonal throughout treatment. Biopsy of a left level 4 cervical lymph node biopsy showed T-ALL and blast cells tested positive for with CD1a, CD2, cCD3, CD4, dim CD5, dim CD7, CD8, and dim TDT. Low levels (0.03%) of precursor T cells were also present in the peripheral blood.

Treatment with a lymphoid based regimen, hyperCVAD, was initiated in combination with ponatinib, (30 mg) a tyrosine kinase inhibitor that has demonstrated activity against leukemia with the *ZMYM2: FGFR1* translocation both in murine models^5^ and in a case study.^6^ At the end of induction (cycles 1A and 1B of hyperCVAD), a BMBx showed a hypercellular marrow with myeloid hyperplasia but with no increase in blasts and no evidence of t(8;13) by FISH, consistent with CR1. Consolidation with HyperCAD+ponatinib was initiated as a bridge to allogeneic hematopoietic stem cell transplantation (HSCT) however the pre-transplant bone marrow biopsy revealed 15.5% of cells were FGFR1-fusion positive. The patient was transitioned from ponatinib to single-agent pemigatinib (13.5 mg daily; Days 1–14 of 21). Due to side effects, including rash and diarrhea, pemigatinib was reduced due to 9 mg (days 1–14 of 21), but again increased to 13.5 mg (continuous), resulting in a precipitous drop from 31% FISH+ cells to 0%. During single agent pemigatinib treatment hemoglobin, RBC count, platelet, and hematocrit levels rose to within the normal range (blue shaded area; Supplemental Figure 1A-D). The patient’s pre-transplant BMBx showed return of measureable residual disease (MRD) positivity with 3.5% FGFR1+ cells by FISH, thus post-transplant pemigatinib maintenance therapy was planned. He underwent a 10/10 matched unrelated donor (MUD) transplant, and remained in an MRD negative remission with no evidence of *FGFR1* translocation by FISH for 8 months. Unfortunately, he then suffered late graft failure and a PET scan demonstrated a single PET avid cervical lymph node, concerning for return of T-ALL, although biopsy demonstrated only necrotic tissue. He underwent treatment with a single cycle of miniMTX/Ara-C +pemigatinib(13.5 mg day 1–14 of 21) as a bridge to a second MUD allogeneic HSCT. Disease staging after his second HSCT demonstrated an MRD negative CR; his day + 60 BMBx showed no evidence of residual T-cell or myeloid neoplasm, FISH with no evidence of his prior FGFR1 fusion and chimerism analysis showed 100% donor in the CD3, CD33 and CD19 cell lines. His day + 100 PET scan showed no evidence of disease. Pemigatinib maintenance (9 mg daily) was initiated after his second HSCT, however it was poorly tolerated. He was hospitalized secondary to pemigatinib side effects including ileitis and hand-and-foot syndrome, and pemigatinib was discontinued permanently. At the time of writing (2 years following his second HSCT), the patient remains in remission with full count recovery and continues to do well.

Primary bone marrow or peripheral blood samples were obtained throughout treatment, and isolated mononuclear cells were exposed *ex vivo* to a panel of drugs as previously described.^7^ Prior to starting treatment, we observed dose-dependent sensitivity of the leukemic cells to bortezomib (Velcade), a proteasome inhibitor previously described as effective for FGFR-overexpressing multiple myeloma^8^ (Figure 1D). FGFR inhibitors, such as axitinib (VEGF/PDGF/FGFRi^9^), ponatinib (ABL/FGFRi), and pemigatinib (FGFRi) were also highly effective (Figure 1E-G; blue curve). Later, mirroring the loss of clinical response to ponatinib, *ex vivo* profiling performed on Day 104 showed reduced sensitivity to ponatinib (Figure 1F; red curve), while sensitivity to pemigatinib remained unchanged (Figure 1G; red curve).

We hypothesized that a mutation might impact ponatinib sensitivity. Sanger sequencing of the *FGFR1* kinase domain performed on available Day 7 and Day 91 samples identified a low-level, acquired nucleotide substitution resulting in a phenylalanine to leucine amino acid change (FGFR1^F686L^ > ZMYM2: FGFR1^F686L^; Supplemental Figure 2A, B). Wild type and mutant *ZMYM2:FGFR1* cDNAs were tagged with V5, and retroviral expression vectors were used to stably express both constructs in Ba/F3 cells. IL-3 withdrawal transformation assays, however, found only the wild type fusion protein (ZMYM2:FGFR1^WT^) to be transforming (Supplemental Figure 2C). The *ZMYM2: FGFR1*^F686L^ kinase domain was PCR amplified from the Ba/F3 cells and confirmed intact with the expected mutation by sequencing. While the wild type and mutant fusion proteins were equally expressed, the mutant protein exhibited a lower molecular weight and anti-phosphotyrosine immunoprecipitation followed by anti-V5 western blotting indicated that the mutant protein was less phosphorylated than the wild type fusion (Supplemental Figure 2D, E). The mutant also showed reduced activation of downstream signaling upregulated by the wild type fusion, including pMEK, pSTAT3 and pAKT (Supplemental Figure 2F). Notably, although distal to the ponatinib binding site, position F686 is located in the αF helix the C-lobe of FGFR, which is ~ 20 residues past the end of activation loop, a region highly conserved among FGFR family members and a key component of the hydrophobic core.^11^ Though the F686L mutant’s lack of transforming capacity precluded formal evaluation of its effect on ponatinib sensitivity, together these data suggest this mutation negatively impacts fusion kinase function and would be unlikely to fully explain the clinical resistance observed in the patient.

To enable broader profiling inhibitor treatment strategies for patients harboring the ZMYM2: FGFR1 fusion, we performed expanded *in vitro* drug screening using IL-3 withdrawn Ba/F3 cells expressing wild-type ZMYM2:FGFR1 (Figure 2A). Drug sensitivity was measured using IC_50_ values, where lower values indicate greater potency. Similar to the *ex vivo* results seen with patient cells, ZMYM2: FGFR1^WT^ cells were sensitive to bortezomib (Velcade) and axitinib,^9^ cediranib (ADZ2171; also inhibits FGFR,^12^ the MEK inhibitor trametinib, and the multi-kinase inhibitor midostaurin. Numerous FGFR inhibitors were found to inhibit ZMYM2: FGFR1^WT^ cell growth, with pemigatinib, infigratinib, AZD4547 and ponatinib showing the most selective impact on the ZMYM2:FGFR1^WT^-expressing cells (Figure 2B, C; Supplemental Figure 3 and Table 1). Ponatinib has previously been shown to prolong survival and reduce spleen size in a murine *ZMYM2:FGFR1* bone marrow transduction model.^5^ Similarly, midostaurin has been show to prolong survival in a murine *ZMYM2:FGFR1* transplantation model and in a *ZMYM2:FGFR1* positive patient^13^ supporting our ex vivo drug screening results.

Given that this patient experienced some dose-dependent toxicities during treatment with approved single-agent FGFR inhibitors, we evaluated lower doses of three FGFR inhibitors (pemigatinib, ponatinib, and infigratinib) in combination with a panel of six tyrosine kinase inhibitors for which we had observed single-agent efficacy in Ba/F3 ZMYM2:FGFR1 cells. Drug matrices (6×6 doses) were performed for each combination on Ba/F3 cells expressing empty vector (MSCV) or ZMYM2:FGFR1^WT^ (top vs bottom dose-response plots, respectively) (Figure 2D, E; Supplemental Figure 4). Although the highest tested doses of some partner drugs (e.g. trametinib 100 nM, midostaurin 500 nM) showed toxicity to the control Ba/F3 cell line, multiple combinations showed a selective and enhanced dose-dependent inhibition of proliferation in ZMYM2:FGFR1 expressing cells. This was most pronounced for combinations pairing the FGFR inhibitors ponatinib, pemigatinib and infigratinib with midostaurin or trametinib (Figure 2D, E; bottom vs top facetted dose-response curves). Furthermore, comparison by both constant drug ratio response curves and by quantification of synergy scores across the full tested dose matrix using the Highest Single Agent (HSA) model^10^ confirmed varying degrees of drug combination-mediated synergistic inhibition of cell growth (Figure 2D, E; heatmap and adjacent dose-response curve).

Western blotting was used to further evaluate the effects of combining midostaurin with increasing ponatinib concentrations on signaling pathways downstream of ZMYM2:FGFR1. In addition to having activity against FGFR1, both ponatinib and midostaurin (previously known as PKC412) are multi-kinase inhibitors which have been used clinically based on their activity against FLT3.^8,12^ Treatment of Ba/F3 ZMYM2:FGFR1 cells with either drug resulted in modest decrease in phospho-FLT3 levels, with greater reduction with the combination. Midostaurin has also previously been shown to decrease phospho-PLCγlevels in ZMYM2/ZNF198: FGFR1-expressing Ba/F3 cells.^13^ Combined treatment with ponatinib and midostaurin showed enhanced decrease in levels of phosphorylated PLCγ, STAT3, and AKT, suggesting that combining midostaurin with ponatinib could be used to further suppress signaling downstream of the fusion (Figure 2F).

Although pemigatinib (Pemazyre) is currently the only FDA approved treatment for adults with relapsed or refractory myeloid/lymphoid neoplasms with FGFR1 rearrangements, together this work underscores the activity of selective FGFR inhibitors, highlights the importance of available next-generation FGFR inhibitors to address resistance, and identifies novel drug combinations that could enhance the impact of FGFR inhibitors, potentially providing an avenue to decrease drug toxicities associated with these treatments in patients with FGFR-fusion driven leukemias.^4^

## Supplementary Material

Supp 1

Supplemental data for this article can be accessed online at https://doi.org/10.1080/28354311.2025.2530229

## Figures and Tables

**Figure 1.**
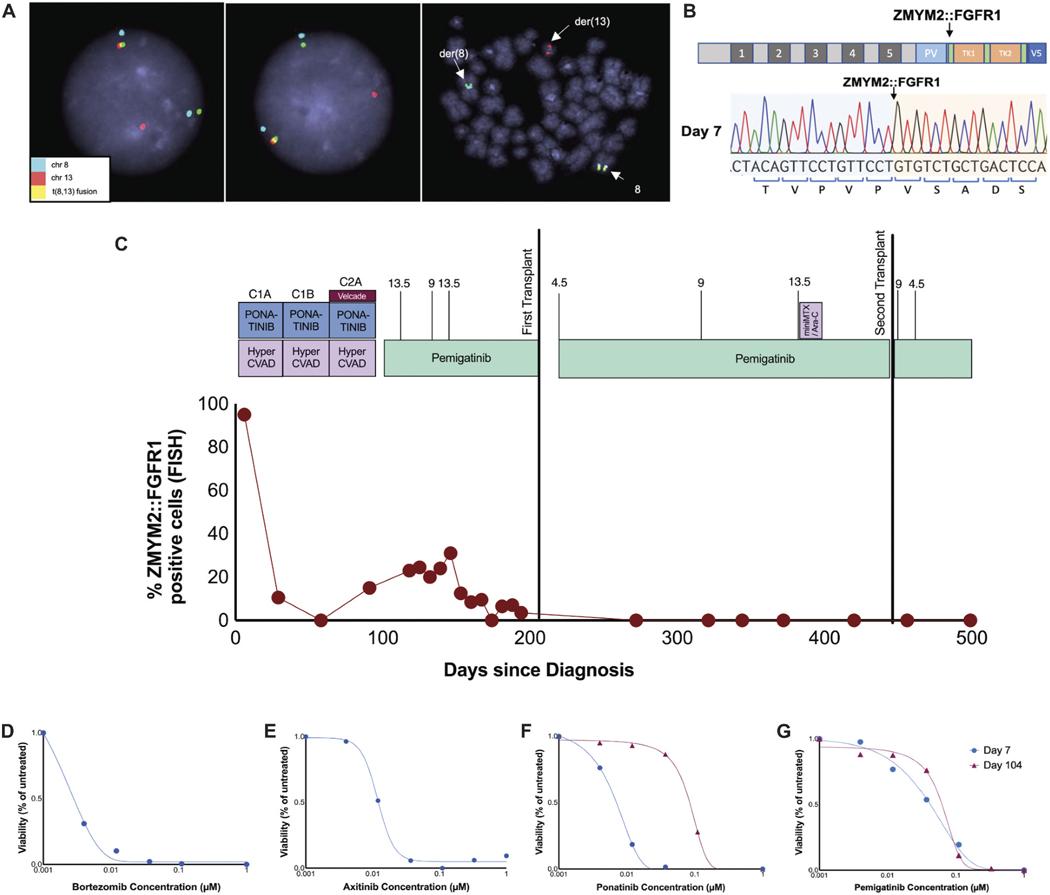
Patient diagnosis and treatment. Schematic overlaying the drug treatments with the patient’s clinical timeline. A. FISH confirmation of *ZMYM2:FGFR1* fusion (yellow) in the patient. *ZMYM2* is on chromosome 8 (blue) and *FGFR1* is on chromosome 13 (red). B. Top: schematic of the ZMYM2:FGFR1 chimeric fusion protein. ZMYM2 region contains 5 MYM domains (gray) and a proline-valine (PV) rich domain (light blue). Breakpoint is indicated by the arrow and the N-terminal FGFR1 region contains the tyrosine kinase domains 1 and 2 (TK1 and TK2; orange). Bottom: the fusion breakpoint was confirmed by Sanger sequencing of the Day 7 sample. C. Timepoints of samples used in the correlate assays are indicated as blue (Day 7), beige (Day 91) and red (Day 104) dots. The patient’s initial diagnosis is designated Day 0, and the first allogeneic transplant occurred around Day 200 and second allogeneic transplant around Day 450 as indicated by the black lines. Fusion levels were monitored by fluorescence in situ hybridization (FISH) throughout treatment and plotted as a percent *ZMYM2:FGFR1* fusion positive cells over time in days (red graph). Dose adjustments of pemigatinib are annotated by numbers above the black lines. D-G. *ex vivo* sensitivity testing of patient samples collected at Day 7 (blue) and/or Day 104 (red) for bortezomib (D), axitinib (E), ponatinib (F) and pemigatinib (G) plotted as the percent viability relative to untreated control cells. Drug concentrations ranged from 0.001 to 1 μM and single curves for each drug were tested due to cell number limitations

**Figure 2.**
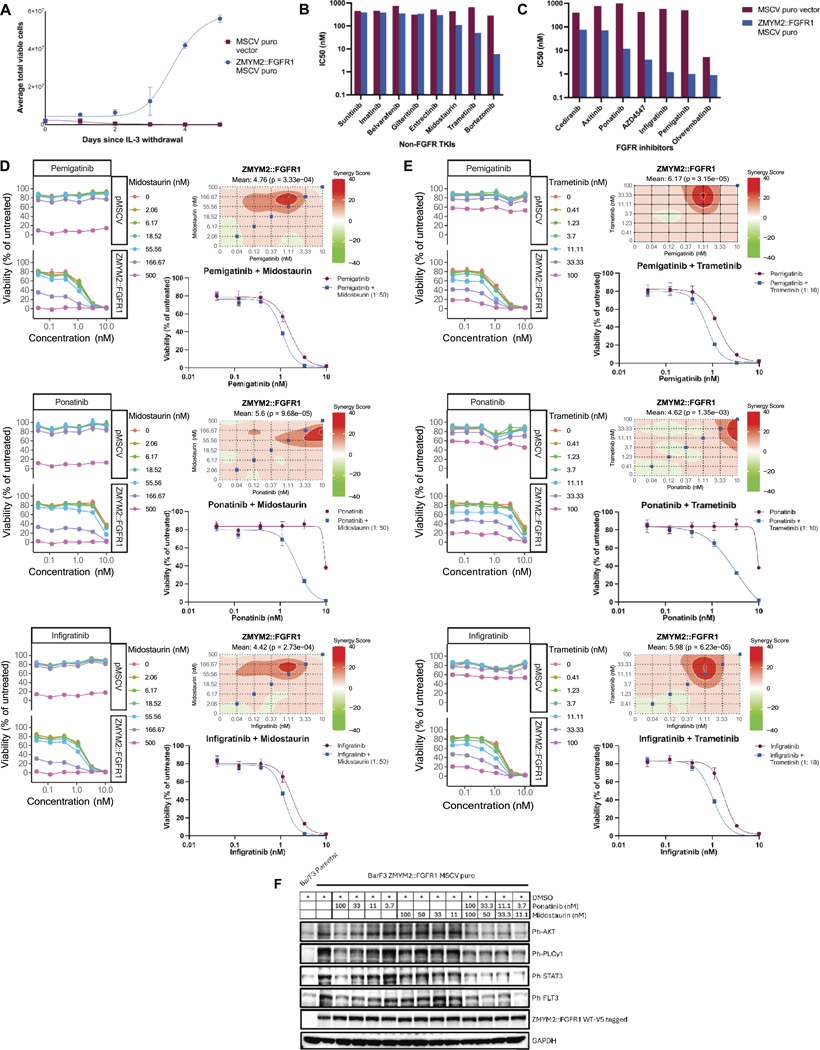
Identification of effective single agents and drug combinations using a ZMYM2:FGFR1 Ba/F3 cell line model. A. The wild type ZMYM2:FGFR1 fusion (ZMYM2:FGFR1^WT^) transformed murine Ba/F3 pro-B cell lines and supported interleukin 3 (IL-3)-independent growth. No growth was observed in the Ba/F3 cells expressing the empty vector (MSCV puro). The average of total viable cells was recorded and plotted over days post-IL-3 withdrawal. B-C. Evaluation of sensitivity of Ba/F3 ZMYM2:FGFR1^WT^ cells to tyrosine kinase inhibitors (TKIs; (B)) and FGFR inhibitors (FGFRi; (C)) that have been FDA-approved or are in advanced stages of approval. Sensitivities are represented as IC_50_ values derived for a 12-point concentration series ranging from 1 μM to 0.005 nM. Cells transduced with MSCV puro empty vector were used as a control. Cells were treated in triplicate and viabilities were measured at 72 hours by MTS assay. D-E. FGFR inhibitors pemigatinib, ponatinib, and infigratinib were used in combination with either midostaurin or trametinib and plotted to display areas of synergy. Pemigatinib and infigratinib were used at concentration ranges of 0.041 to 10 nM, and ponatinib was used at a concentration range of 0.205 to 50 nM in combination with either (D) midostaurin (2.057 to 500 nM) or (E) trametinib (0.411 to 100 nM). Combination dose response curves compare ZMYM2:FGFR1^WT^ sensitivities (bottom) with empty vector control (MSCV; top) at increasing concentrations. Heatmaps of synergy scores were calculated using the highest single agent (HSA) model (^10^; top right plot) across the full matrix of combined doses tested for each drug pair. Dose-response curves for the ZMYM2:FGFR1^WT^ cells using only constant ratio dose combinations of FGFRi with either midostaurin or trametinib (indicated as blue squares on synergy plots) are plotted in comparison to sensitivities for single-agent pemigatinib, ponatinib, or infigratinib (bottom right graphs of each panel). F. Ponatinib combined with midostaurin inhibits activation of downstream signaling pathways. Cells were serum starved overnight and treated with either a DMSO vehicle control, each single agent (midostaurin or ponatinib), or a combination of ponatinib and midostaurin at the concentrations indicated for 4 hours at 37°C. Western blots were performed to evaluate downstream signaling proteins: ph-AKT, ph-PLCy1, ph-Stat3, and ph-FLT3. V5 antibodies were used to detect the ZMYM2:FGFR1^WT^ protein. GAPDH was used as a loading control

## References

[1] Khoury JD, Solary E, Abla O, Akkari Y, Alaggio R, Apperley JF, Bejar R, Berti E, Busque L, Chan JKC, The 5th edition of the world health organization classification of Haematolymphoid tumours: myeloid and Histiocytic/Dendritic neoplasms. Leukemia. 2022 Jul. 36(7):1703–19. doi: 10.1038/s41375-022-01613-1. 35732831 PMC9252913

[2] Jackson CC, Medeiros LJ, Miranda RN. 8p11 myeloproliferative syndrome: a review. Hum Pathol. 2010 Apr. 41(4):461–76. doi: 10.1016/j.humpath.2009.11.003. 20226962

[3] Barnes EJ, Leonard J, Medeiros BC, Druker BJ, Tognon CE. Functional characterization of two rare BCR-FGFR1(+) leukemias. Cold Spring Harb Mol Case Stud. 2020 Apr. 6(2):a004838. doi: 10.1101/mcs.a004838. PMC7133745 31980503

[4] Krook MA, Reeser JW, Ernst G, Barker H, Wilberding M, Li G, Chen H-Z, Roychowdhury S. Fibroblast growth factor receptors in cancer: genetic alterations, diagnostics, therapeutic targets and mechanisms of resistance. Br J Cancer. 2021 Mar. 124 (5):880–92. doi: 10.1038/s41416-020-01157-0. 33268819 PMC7921129

[5] Ren M, Qin H, Ren R, Cowell JK. Ponatinib suppresses the development of myeloid and lymphoid malignancies associated with FGFR1 abnormalities. Leukemia. 2013 Jan. 27(1):32–40. doi: 10.1038/leu.2012.188. 22781593 PMC3629706

[6] Khodadoust MS, Luo B, Medeiros BC, Johnson RC, Ewalt MD, Schalkwyk AS, Bangs CD, Cherry AM, Arai S, Arber DA, Clinical activity of ponatinib in a patient with FGFR1-rearranged mixed-phenotype acute leukemia. Leukemia. 2016 Apr. 30(4):947–50. doi: 10.1038/leu.2015.136. 26055304 PMC5369353

[7] Tyner JW, Tognon CE, Bottomly D, Wilmot B, Kurtz SE, Savage SL, Long N, Schultz AR, Traer E, Abel M, Functional genomic landscape of acute myeloid leukaemia. Nature. 2018 Oct. 562 (7728):526–31. doi: 10.1038/s41586-018-0623-z. 30333627 PMC6280667

[8] Guan M, Zhu L, Somlo G, Hughes A, Zhou B, Yen Y. Bortezomib therapeutic effect is associated with expression of FGFR3 in multiple myeloma cells. Anticancer Res. 2009 Jan. 29(1):1–9. 19331127

[9] Bakri SJ, Lynch J, Howard-Sparks M, Saint-Juste S, Saim S. Vorolanib, sunitinib, and axitinib: a comparative study of vascular endothelial growth factor receptor inhibitors and their anti-angiogenic effects. PLoS One. 2024;19(6):e0304782. doi: 10.1371/journal.pone.0304782. PMC11149885 38833447

[10] Berenbaum MC. What is synergy? Pharmacol Rev. 1989 June. 41(2):93–141. doi: 10.1016/S0031-6997(25)00026-2. 2692037

[11] Dai S, Zhou Z, Chen Z, Xu G, Chen Y. Fibroblast growth factor receptors (FGFRs): structures and small molecule inhibitors. Cells. 2019 June 18. 8(6):614. doi: 10.3390/cells8060614. 31216761 PMC6627960

[12] Wedge SR, Kendrew J, Hennequin LF, Valentine PJ, Barry ST, Brave SR, Smith NR, James NH, Dukes M, Curwen JO, AZD2171: a highly potent, orally bioavailable, vascular endothelial growth factor receptor-2 tyrosine kinase inhibitor for the treatment of cancer. Cancer Res. 2005 May 15. 65(10):4389–400. doi: 10.1158/0008-5472.CAN-04-4409. 15899831

[13] Chen J, Deangelo DJ, Kutok JL, Williams IR, Lee BH, Wadleigh M, Duclos N, Cohen S, Adelsperger J, Okabe R, PKC412 inhibits the zinc finger 198-fibroblast growth factor receptor 1 fusion tyrosine kinase and is active in treatment of stem cell myeloproliferative disorder. Proc Natl Acad Sci USA. 2004 Oct 5. 101(40):14479–84. doi: 10.1073/pnas.0404438101. 15448205 PMC521956

