# Supplementary material for: Identifying Drug Combination Strategies for ZMYM2: FGFR1 Fusion Positive Leukemia
Source: Precis Oncogenom. Author manuscript; Available in PMC 2025 Aug 27. (PMC12379768; doi:10.1080/28354311.2025.2530229)
Supplement: Supp 1 [file NIHMS2095487-supplement-Supp_1.docx]

1 SUPPLEMENTAL DOCUMENT:

2

3 Identifying Drug Combination Strategies for ZMYM2::FGFR1 Fusion Positive Leukemia

4

1. Ariane Huang^1*^, Sofia R. Beer^1*5^, Christopher A. Eide^1,2^, Brian J. Druker^1,2,4^, Jeffrey W. Tyner^1,2,3^,
2. Jessica Leonard^1,2^ and Cristina E. Tognon^1,^ ^4^

7

1. ^1^Knight Cancer Institute, Oregon Health & Science University, Portland, OR
2. ^2^Division of Hematology & Medical Oncology, Department of Medicine, Oregon Health & Science
3. University, Portland, OR
4. ^3^Department of Cell, Developmental, and Cancer Biology, Oregon Health & Science University,
5. Portland, OR, USA
6. ^4^Division of Oncological Sciences, Department of Medicine, Oregon Health & Science University,
7. Portland, OR
8. ^5^Loyola University Chicago Stritch School of Medicine, Maywood, IL

16

17

18 * Denotes equal contribution

19

20 **^#^CORRESPONDENCE:** Cristina E. Tognon, Oregon Health & Science University, 3181 SW Sam

21 Jackson Park Rd, Mail Code: KR-HEM, Portland, OR 97239; E-mail: [tognon@ohsu.edu](mailto:tognon@ohsu.edu)

22

23

24 **RUNNING TITLE:** ZMYM2::FGFR1 inhibitor sensitivity

25

26

27

28 **KEYWORDS:** ZMYM2::FGFR1, AML, Leukemia, FGFR inhibitors, Pemigatinib, Fusion Protein

29

30

31

32

33

34 **MATERIAL & METHODS:**

35

1. *Cell lines*
2. Cell lines were maintained at 37°C in 5% CO2. Cells were cultured in the following media:
3. HEK 293T/17 cells (ATCC, Manassas, VA): DMEM (Life Technologies Inc., Carlsbad, CA)
4. supplemented with 10% FBS (Atlanta Biologicals, Flowery Branch, GA), 2% L-glutamine, 1%
5. penicillin/streptomycin (Life Technologies Inc.), and 0.1% amphotericin B (HyClone, South Logan,
6. UT); Ba/F3 cells: RPMI1640 (Life Technologies Inc.) supplemented with 10% FBS, 2%
7. L- glutamine, 1% penicillin/streptomycin, 0.1% amphotericin B as stated above, and 15%
8. WEHI3B-conditioned medium (a source of IL-3). Cells were kept in culture no longer than a month
9. at a time, and all cell lines were authenticated by extensive functional and genetic analysis in our
10. lab.

46

1. *Sequencing*
2. RNA obtained from primary leukemia mononuclear cells (QIAGEN RNeasy Mini Kit)
3. served as template for cDNA synthesis as recommended by the manufacturer (Superscript Vilo
4. Kit, Thermo Fisher Scientific). Amplification of the *ZMYM2::FGFR1* fusion breakpoint was
5. performed by hot start PCR (Q5 Hot Start High-Fidelity 2x Master Mix (M0494S)) using forward
6. and reverse primers: (For1:ctgtgtatatcccagttcctatgcac; Rev2:gcaggacaccaggtccttggag). PCR
7. products were electrophoresed on a 1% agarose gel to purify the PCR product (QIAquick PCR
8. Purification Kit; QIAGEN) and subjected to conventional Sanger sequencing using the following
9. primers: For1: 5’-ctgtgtatatcccagttcctatgcac-3’; For2: 5’-ggaatactgctacaaccccagc-3’; For3: 5’-
10. cgagctgtacatgatgatgcg-3’; Rev1: 5’-gagggttacagctgacggtg-3’; and Rev2: 5’-
11. gcaggacaccaggtccttggag-3’. Sanger sequencing of the *ZMYM2::FGFR1* fusion was performed by
12. Eurofins.
13. *Cytogenetics and Fluorescence In Situ Hybridization*
14. Standard trypsin and Wright (GTW)-banded karyotype analysis and FISH techniques were
15. performed following standard clinical protocols. Briefly, 200 cells were analyzed for disruptions in
16. *FGFR1* and interphase nuclei were probed using the *FGFR1* separation probe (Cytocell), which
17. comprised two *FGFR1* flanking probes. One probe covered 272 kb on one side of the *FGFR1*
18. gene, and the other covered 267 kb on the other side of the gene.

65

1. *Generation of ZMYM2::FGFR1^WT^ and FGFR1^F686L^ Cell Lines and IL-3 withdrawal assay*
2. C-terminal V5-tagged ZMYM2::FGFR1^WT^ and FGFR1^F686L^ in MSCV IRES-puro retroviral
3. vectors were generated by Vector Builder. To produce murine retrovirus, HEK 293T/17 cells were
4. co-transfected with FuGENE 6 (Promega, Madison, WI), EcoPac helper packaging plasmid, and
5. MSCV ZMYM2::FGFR1^WT^ or MSCV FGFR1^F686L^ IRES-puro retroviral vector or MSCV puro empty
6. vector control. Retroviral supernatants were harvested 48 hours after transfection. Stable Ba/F3
7. empty vector, ZMYM2::FGFR1^WT^, or ZMYM2::FGFR1^F686L^ expressing cell lines were generated
8. by infection of 3 x 10^6^ cells with 1 mL of retroviral supernatant followed by spinoculation with
9. polybrene at 2500 rpm for 90 minutes. Infected Ba/F3 cells were then selected using 2 μg/mL
10. puromycin (ThermoFisher Scientific, Waltham, MA). Ba/F3 cells expressing MSCV puro,
11. ZMYM2::FGFR1^WT^, or FGFR1^F686L^ were washed three times in PBS (Life Technologies Inc) to
12. remove all traces of IL-3-containing WEHI3B-conditioned media. Cells were then suspended at a
13. density of 5 x 10^5^ cells per mL and viable cells were counted on a Guava Muse Cell Analyzer
14. (Luminex Inc, Austin, TX) every other day and divided as necessary.

80

1. *Western Blotting*
2. Parental Ba/F3 cells and Ba/F3 expressing MSCV puro, ZMYM2::FGFR1^WT^, or
3. ZMYM2::FGFR1^F686L^ were serum starved overnight in RPMI1640 media supplemented with 0.1%
4. bovine serum album 2% L-glutamine, 1% penicillin, streptomycin, and 0.1% amphotericin B. Cells
5. were collected at 400 RCF for 5 minutes at 4^o^C and lysed in 100 μL of Cell Lysis Buffer (Cell
6. Signaling Technologies Inc., Danvers MA) containing a Complete Mini Protease Inhibitor Cocktail
7. Tablet, Phosphatase Inhibitor Cocktail 2, and Phenylmethanesulfonylfluoride (PMSF) solution
8. (Sigma-Aldrich Inc., St Louis, MO) and agitated with rotary action for 30 minutes at 4^o^C. Lysates
9. were clarified by centrifugation at 14,000 x g, at 4^o^C for 20 minutes, and quantified with
10. bicinchoninic acid (BCA) assay (ThermoFisher Scientific Inc.). 75 μg of each protein lysate was
11. loaded on 4-15% Criterion Tris-HCl Protein Gels (Bio-Rad Inc.) and run for 1 hour at 180V.
12. Following overnight transfer at 25V, the membrane was blocked in 5% BSA for 1 hour at room
13. temperature and incubated overnight with primary antibodies (**Supplemental Table 2**) at 4^o^C.
14. The membranes were washed and probed with goat anti- rabbit IgG or anti-mouse IgG Polyclonal
15. Antibody (Horseradish Peroxidase; 1:15,000; Promega Inc.) and imaged with the Bio-Rad
16. ChemiDocTM MP Imaging System (Bio-Rad Inc. Richmond, CA).

97

1. *Ex vivo Sensitivity Assays*
2. Small-molecule inhibitors used in this study are listed in **Supplemental Table 1**. Inhibitors were
3. purchased from Selleck Chemicals (Houston, TX) and MedChemExpress (Monmouth Junction,
4. NJ) and were reconstituted in DMSO and stored at -80^o^C. Ba/F3 MSCV puro empty vector and
5. ZMYM2::FGFR1^WT^ cell lines were seeded in 384-well plates using the Multidrop™ Combi
6. Reagent Dispenser (ThermoFisher Scientific Inc.) at a density of 800 cells/well in 50 µL of
7. RPMI1640 media supplemented with 10% fetal bovine serum, 2% L-glutamine, 1% penicillin,
8. streptomycin, and 0.1% amphotericin B. Ba/F3 empty vector expressing cells were grown in
9. media containing additional 15% WEHI3B-conditioned media as a source of IL-3. Inhibitors were
10. dispensed using the HP D300e Digital Dispenser (Tecan, Mannedorf, Switzerland) at increasing
11. concentrations, with less than 0.1% DMSO in all wells. All conditions were performed in triplicate
12. and treated for 72 hours at 37°C in 5% CO2. Cell viability was quantified using an MTS-based
13. tetrazolium assay (CellTiter96 Aqueous One solution; Promega) and absorbances (490 nm) were
14. read 2 hours after the addition of the MTS reagent using a Bio Tek Synergy 2 plate reader (Bio
15. Tek, Winooski, VT). Analysis, normalization of MTS absorbances of treated wells to untreated to
16. generate combination graphs were performed using R, with synergy and inhibition plots generated
17. using the SynergyFinder package. GraphPad Prism software was used to perform regression
18. curve fit analyses of the normalized data to determine IC50 and AUC values.

116

117

118

119

120

121

122

123

124

125

126

127

128

129

130

131

132

133

134

135

136

**
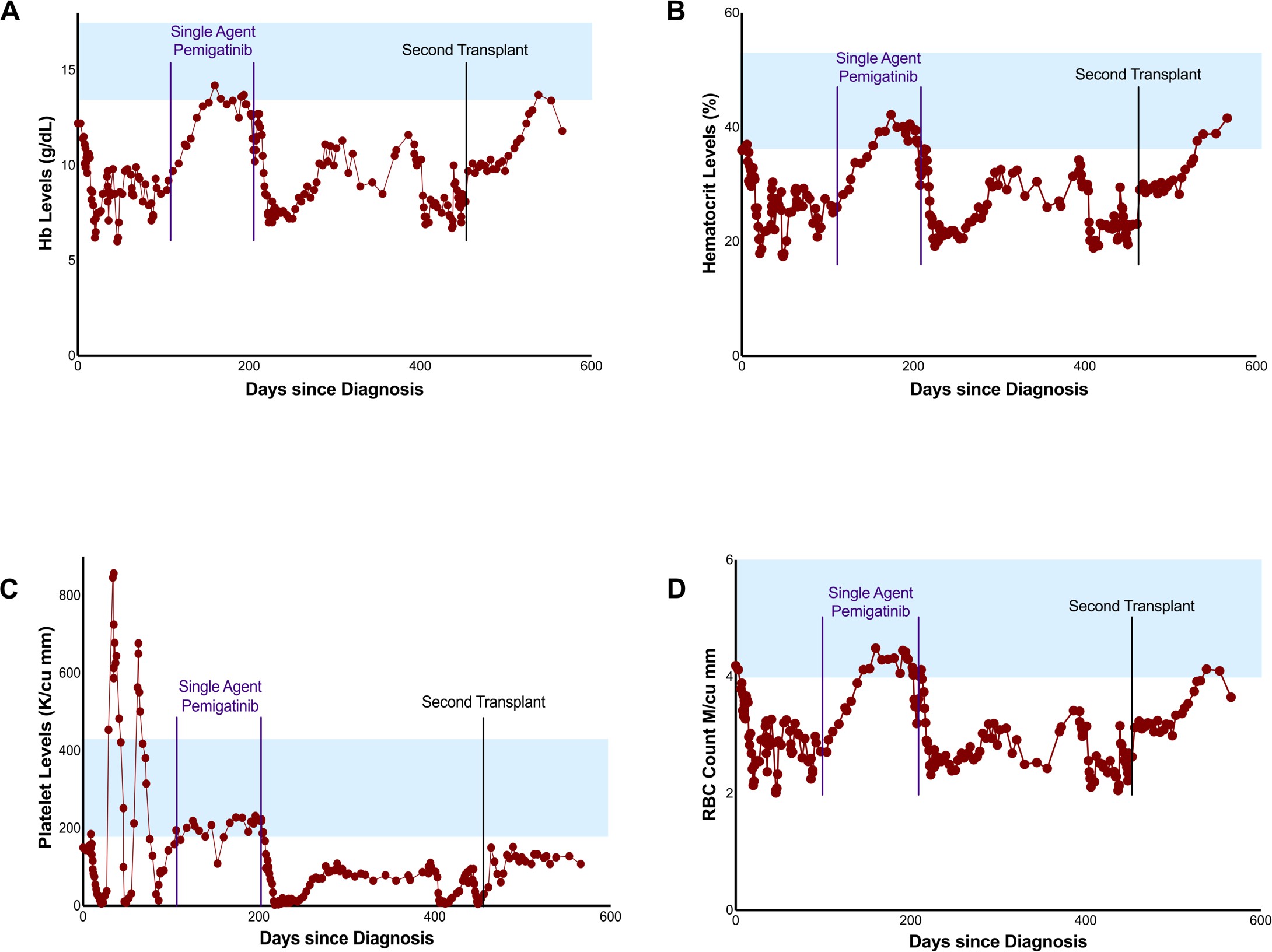
**137 **SUPPLEMENTAL FIGURES:**

138

# Supplemental Figure 1: Single agent pemigatinib treatment resulted in normalization of

1. **patient blood constituent levels. A-D**. Red blood cell (RBC) counts, hemoglobin (Hb), platelet,
2. and hematocrit levels were plotted over time (red). The blue boxes highlight the normal ranges,
3. purple bars indicate time from when the patient initially started on pemigatinib single agent
4. treatment to the first transplant. Black bar indicates the time of the second transplant.

144

145

146

147

148

149


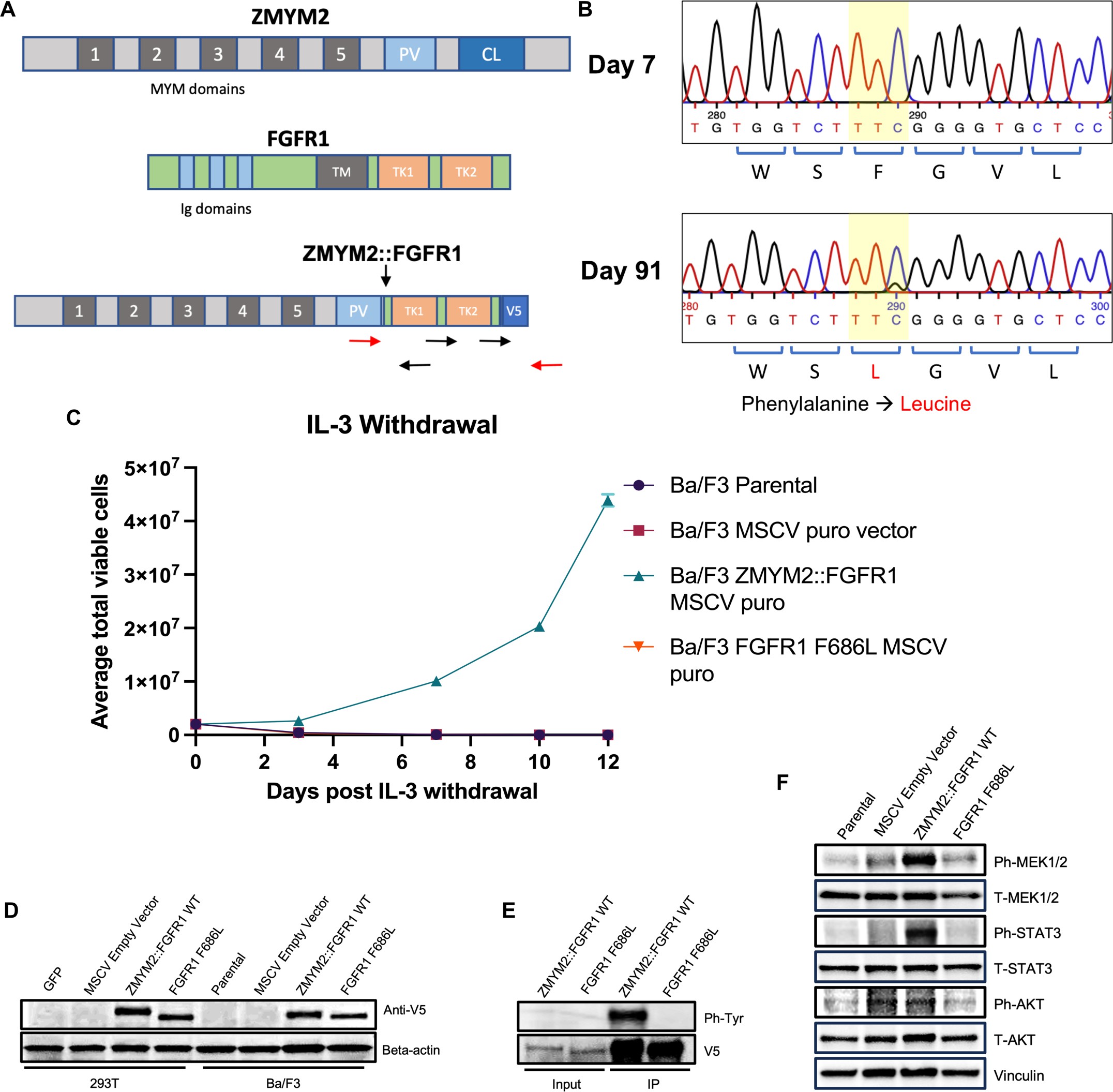
150

# Supplemental Figure 2: Point mutation detected in ZMYM2::FGFR1 in the Day 91 sample

1. **impacts kinase activity**. **A**. Schematic of the molecular domains of ZMYM2 and FGFR1. The
2. fusion protein contains the 5 zinc finger domains (MYM1) and the proline-valine (PV) portion of
3. ZMYM2. The FGFR1 kinase domain is found in the C-terminus of the fusion protein and the
4. breakpoint is indicated by the black vertical arrow. Fusion constructs were engineered with a C-
5. terminal V5-tag. Primers used in this study are indicated by arrows underneath the schematic.
6. Red indicates the primers used to amplify the *FGFR1* portion of the fusion from the Day 7 and
7. Day 91 samples. Note: No sample was available for Day 104. Black arrows indicate additional
8. sequencing primers. **B.** Sanger sequencing results from Days 7 and 91 samples identified a
9. phenylalanine to lysine amino acid change in FGFR1 at position 686. **C**. Retroviral constructs of
10. ZMYM2::FGFR1^WT^ and FGFR1^F686L^ were stably expressed in Ba/F3 cells. The experiment was
11. performed in triplicate and the averaged total viable cells were recorded over days post IL-3
12. withdrawal. **D**. HEK293/T17 and Ba/F3 cells expressing empty vector MSCV puro,
13. ZMYM2::FGFR1^WT^, or FGFR1^F686L^ were analyzed by western blotting and compared to
14. untransfected Ba/F3 cells. V5 antibody was used to detect the fusions and beta-actin was used
15. as loading control. **E**. Co-immunoprecipitation of ZMYM2::FGFR1^WT^ and FGFR1^F686L^ expressing
16. Ba/F3s. Input lysate showed equal levels of fusion protein expression. Anti-phosphotyrosine
17. antibodies were used to evaluate the phosphorylation levels and V5 was used to probe for the
18. fusions. **F**. Ba/F3 cells alone or Ba/F3 cells expressing MSCV puro empty vector,
19. ZMYM2::FGFR1^WT^ or FGFR1^F686L^ were serum starved overnight and evaluated for downstream
20. signaling by western blot analysis. Western blots were probed with phospho MEK1/2, STAT3,
21. and AKT, and total MEK1/2, STAT3, and AKT. Vinculin was used as a loading control.


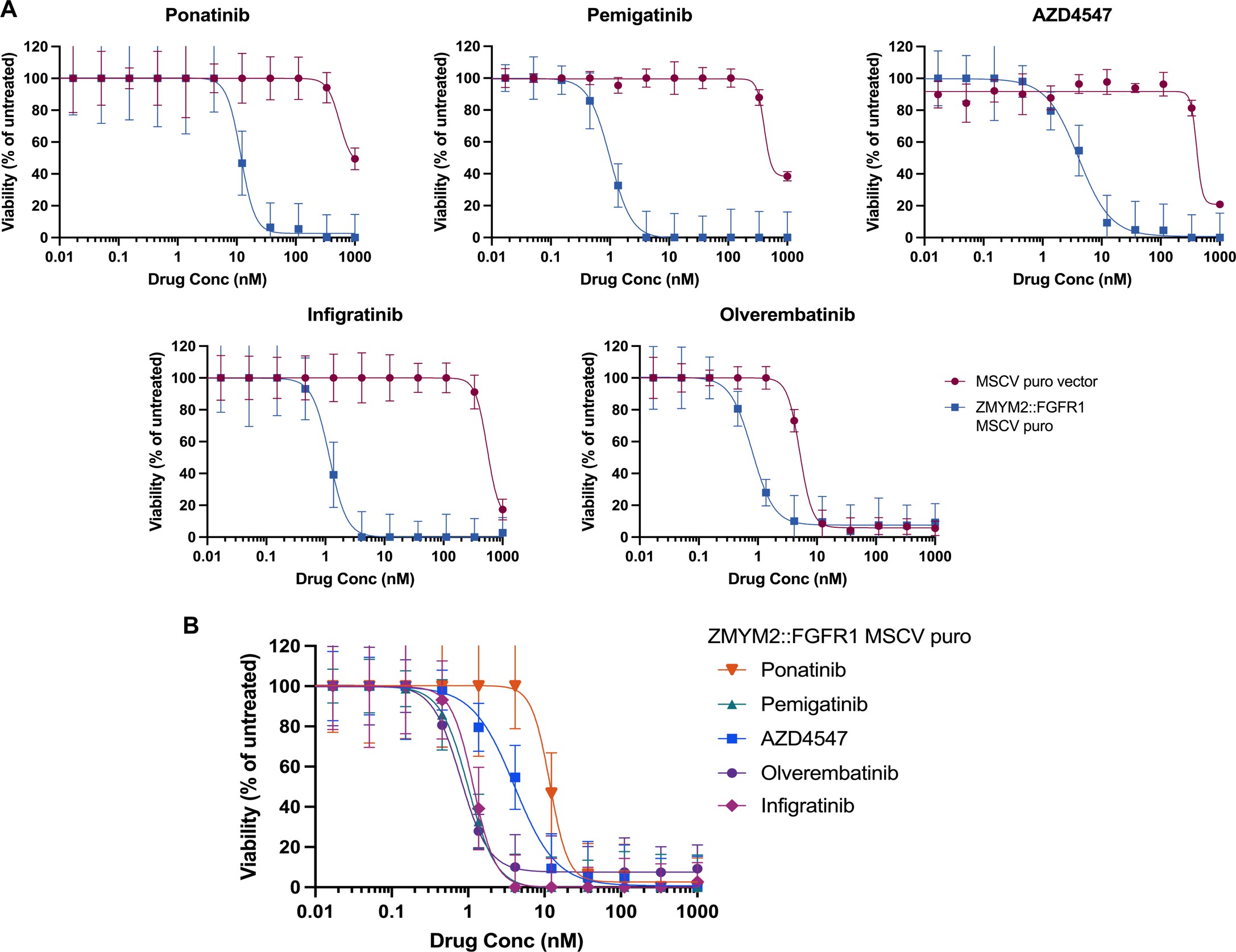
173

# Supplemental Figure 3: Comparison of Ba/F3 ZMYM2::FGFR1^WT^ cell sensitivities to FGFR

1. **inhibitors. A**. Viability as a percentage of untreated cells for MSCV puro vector expressing (red
2. curve) and ZMYM2::FGFR1^WT^ expressing (blue curve) Ba/F3 cells when exposed to increasing
3. concentrations of the following FGFR inhibitors: ponatinib, pemigatinib, AZD4547, infigratinib,
4. and olverembatinib. The dose ranges of inhibitors tested are described in **Figure 2B&C**. MSCV
5. puro vector was tested as a control and plotted in red. **B**. Drug sensitivity curves for Ba/F3
6. ZMYM2::FGFR1 cells to each FGFR inhibitor from **Figure 3A** are overlayed to compare drug
7. activity.

182

183

184

185
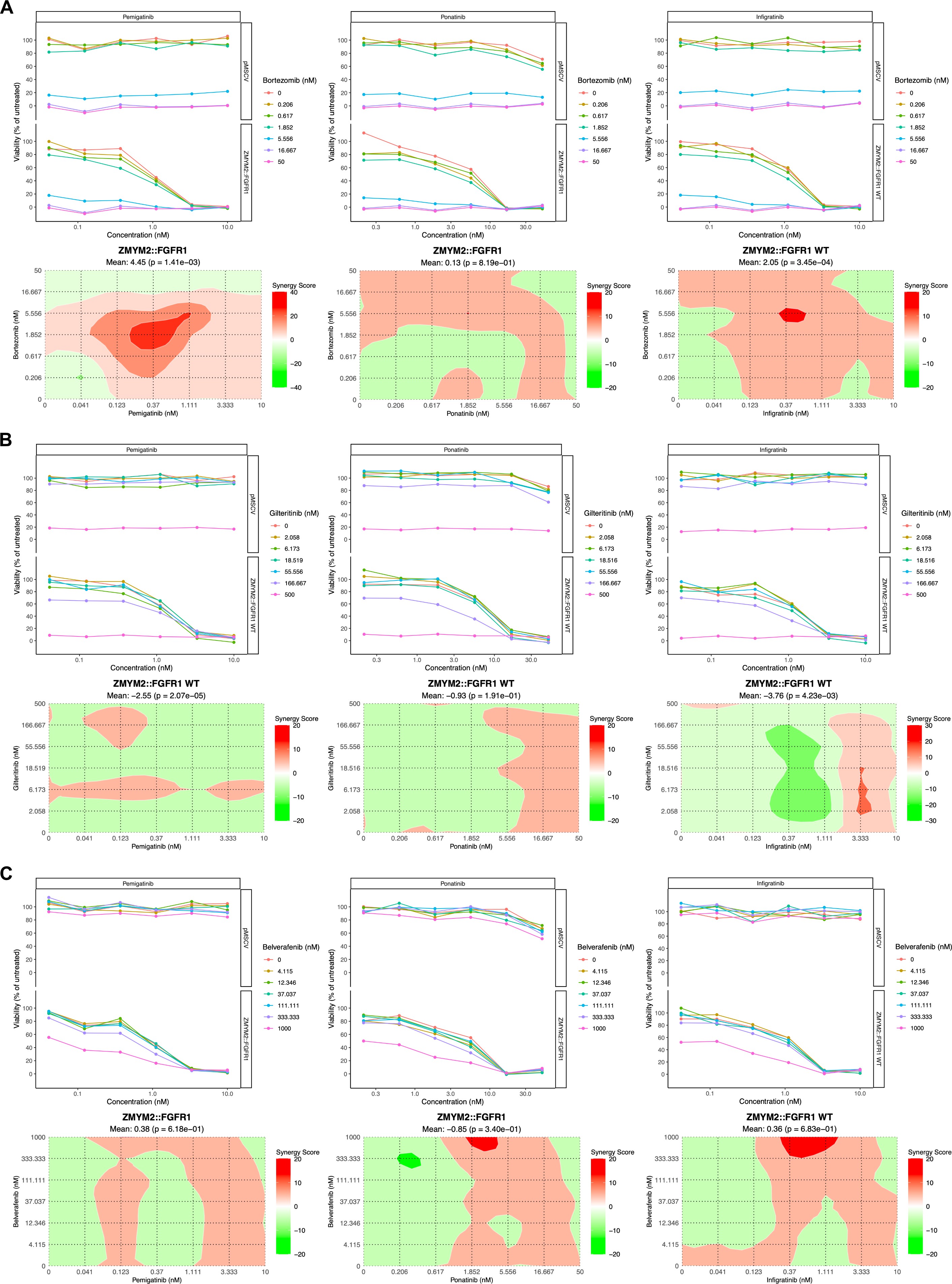


186

187

188

189

190

191

192

193

194

195

196

197

198

199

200

201

202

203

204

**Supplemental Figure 4. Other TKI and FGFRi combination studies in ZMYM2::FGFR1^WT^ Ba/F3 cells.** Ex vivo combination studies of FGFR inhibitors pemigatinib, ponatinib, and infigratinib (used at concentrations described in Figure 2) were evaluated for synergistic activity with the TKIs bortezomib (0.206 to 50 nM), gilteritinib (2.058 to 500 nM), and belverafenib (4.115 to 1000 nM) (**A-C**). Cells were treated in triplicate and viabilities were measured at 72 hours and calculated as a percentage of untreated cells. Combination dose curves were generated using R and highest single agent (HSA) synergy plots were generated using the synergyfinder package in R, as described in **Figure 2**.

**SUPPLEMENTAL TABLES:**

**Supplemental Table 1: Effects of FGFR and non-FGFR inhibitors on the ZMYM2::FGFR1 fusion expressing Ba/F3 cells.** Control MSCV puro Ba/F3 cells and Ba/F3 cells transformed with ZMYM2::FGFR1^WT^ were subjected to 12-point drug concentration series. AUCs (% of max) and IC50 (nM) were calculated (as described in **Figures 2B and C**) for each cell line and drug.

**Supplemental Table 1**

| **Inihibitors** | | **AUC (% of max)** | | **IC50 (nM)** | |
| --- | --- | --- | --- | --- | --- |
|  |  | **MSCV puro vector** | **ZMYM2::FGFR1**  **MSCV puro** | **MSCV puro vector** | **ZMYM2::FGFR1**  **MSCV puro** |
| **FGFRi** | Olverembatinib | 59.3 | 45.6 | 5.3 | 0.9 |
|  | Pemigatinib | 95.7 | 42.5 | 499.1 | 1.0 |
|  | Infigratinib | 95.4 | 44.0 | 581.6 | 1.2 |
|  | AZD4547 | 87.7 | 54.6 | 422.2 | 4.1 |
|  | Ponatinib | 97.2 | 64.4 | 971.3 | 11.9 |
|  | Axitinib | 93.2 | 69.8 | 764.5 | 70.1 |
|  | Cediranib | 93.0 | 74.8 | 397.2 | 75.7 |
| **non-FGFR TKIs** | Bortezomib | 89.1 | 60.4 | 281.0 | 5.8 |
|  | Trametinib | 97.0 | 75.2 | 653.8 | 49.7 |
|  | Midostaurin | 92.4 | 80.7 | 433.4 | 110.9 |
|  | Entrectinib | 94.9 | 89.7 | 512.9 | 297.1 |
|  | Gilteritinib | 89.5 | 90.8 | 311.1 | 337.5 |
|  | Belvarafenib | 90.9 | 83.8 | 751.4 | 348.2 |
|  | Imatinib | 95.0 | 90.1 | 453.4 | 383.9 |
|  | Sunitinib | 94.8 | 82.8 | 442.6 | 389.4 |

205

206

207

**Supplemental Table 2: Antibodies used for western blotting.**

| **Target** | **Antibody product #** | **Vendor** | **Clone** | **Species** | **Antibody Dilution** | **Predicted Size (kDa)** |
| --- | --- | --- | --- | --- | --- | --- |
| Stat3 | 9139S | Cell Signaling | 123H6 | M | 1:1000 | 79, 86 |
| MEK 1/2 | 8727S | Cell Signaling | D1A5 | R | 1:1000 | 45 |
| AKT | 9272S | Cell Signaling | - | R | 1:1000 | 60 |
| PLC𝛾2 | 3872S | Cell Signaling | Y783 | R | 1:500 | 150 |
| PI3K | 4292S | Cell Signaling | p85 | R | 1:1000 | 85 |
| ANTI-V5 | 46-0705 | Invitrogen | SV5-Pk1 | M | 1:5000 | 130 |
| P-Stat3 | 9131S | Cell Signaling | Y705 | R | 1:500 | 79, 86 |
| P-MEK  1/2 | 9154S | Cell Signaling | S217/221 | R | 1:500 | 45 |
| P-AKT | 9271L | Cell Signaling | S473 | R | 1:500 | 60 |
| P-PLC𝛾1 | 2821S | Cell Signaling | Y783 | R | 1:500 | 155 |
| P-Tyr | 05-321 | Sigma Aldich | 4G10 | M | 1:500 | - |
| Ph-FLT3 | 3461S | Cell Signaling | Tyr591 | R | 1:500 | 130, 160 |
| Alpha- tubulin | 3873S | Cell Signaling | DM1A | M | 1:5000 | 52 |
| Beta-actin | 3700S | Cell Signaling | 8H10D10 | M | 1:5000 | 45 |
| GAPDH | 5174S | Cell Signaling | D16H11 | R | 1:5000 | 37 |
| Vinculin | 13901S | Cell Signaling | E1E9V | R | 1:5000 | 124 |
